# Supplementary material for: Precise targeting for 3D cryo-correlative light and electron microscopy volume imaging of tissues using a FinderTOP
Source: Commun Biol. 2023 May 11;6:510. doi: 10.1038/s42003-023-04887-y (PMC10175257; doi:10.1038/s42003-023-04887-y)
Supplement: Supplementary file 2 — Supporting Information [file 42003_2023_4887_MOESM2_ESM.pdf]

Supporting Information for

Precise targeting for 3D cryo-correlative light and electron microscopy volume imaging of tissues using a FinderTOP

Marit de Beer[$1,2], Deniz Daviran[$1,2], Rona Roverts[$1,2], Luco Rutten[2], Elena Macías-Sánchez[2,3], Juriaan R. Metz[4], Nico Sommerdijk[1,2*], Anat Akiva[1,2*]

[1] Electron Microscopy Center, Radboud Technology Center Microscopy, Radboud University Medical Center, Nijmegen, the Netherlands.

[2] Department of Medical Biosciences, Radboud University Medical Center, Nijmegen, the Netherlands.

[3] Department of Stratigraphy and Paleontology, University of Granada, Granada, Spain

[4] Department of Animal Ecology and Physiology, Radboud Institute for Biological and Environmental Sciences, Faculty of Science, Radboud University, Nijmegen, The Netherlands

$ These authors contributed equally.

* Corresponding authors: nico.sommerdijk@radboudumc.nl; anat.akiva@radboudumc.nl

Figure S1. Overlay of cryoFIB/SEM and CACM in x-z view. (a-c) comparison of the x-z view in cryoFIB/SEM and CACM showing the optical aberrations introduced by refraction at the air-ice interface. (a) cryo-FIB/SEM slice. (b) x-z reflection image from resliced CACM stack corresponding to white dashed box in (a), showing the osteoblasts (yellow) and mitochondria (magenta). Arrow indicates the air-ice interface. Osteoblasts appear too close to the air-ice interface; mitochondria appear outside locations where cells are present. (c) x-y reflection image of the first z-position where the air-ice interface is detected, corresponding to the arrow in (b). (d-f) Overlay of cryoFIB/SEM and CACM data after wavelength specific z-correction. 640 nm reflection is used to indicate air-ice interface. To match with the cryoFIB/SEM the overlay of the 488 nm (osteoblasts) channel was stretched with 155% in the z-direction, the overlay of the 561nm (mitochondria) with 180%. (d) overlay with stretched 488nm image (e) with stretched 561nm image (f) with both 488nm and 561nm images.

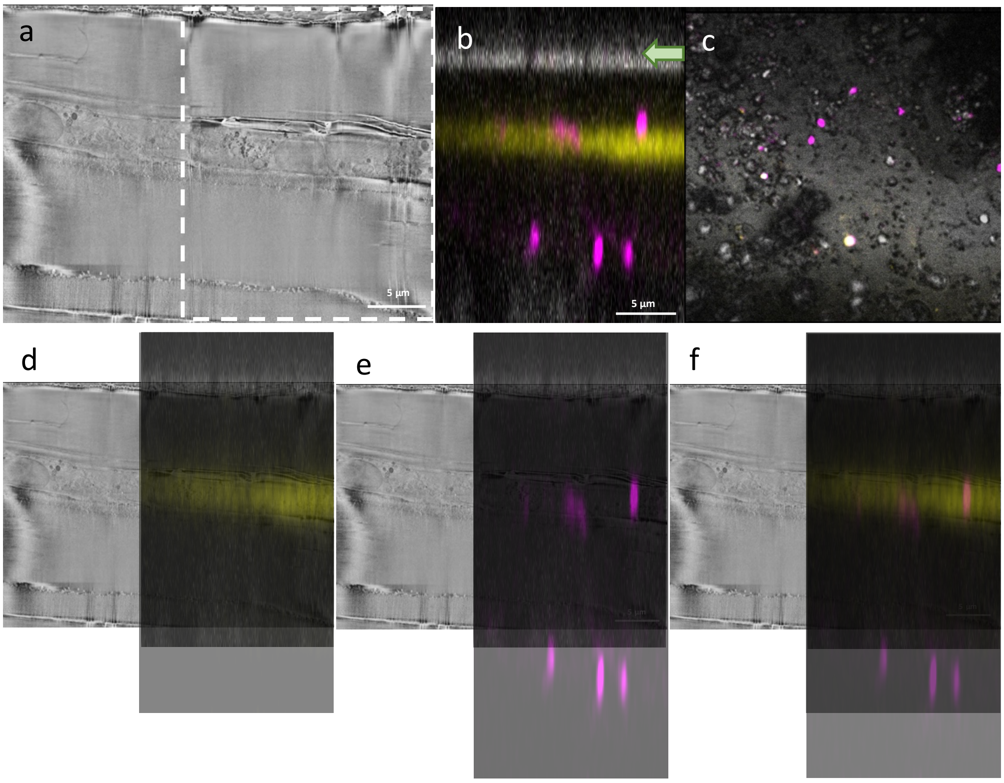

Figure S2. Unprocessed secondary electron image in cryo-FIB/SEM. Raw secondary electron image (InLense detector) in cryo-FIB/SEM before processing showing low-signal-to-noise ratios (low contrast) due to the low electron dose used for imaging (avoiding electron beam damage). Image also contains the typical FIB/SEM acquisition artifacts including vertical striping ("curtaining") due to uneven FIB milling, horizontal striping due to charging, and uneven illumination due to inhomogeneous detector sensitivity.

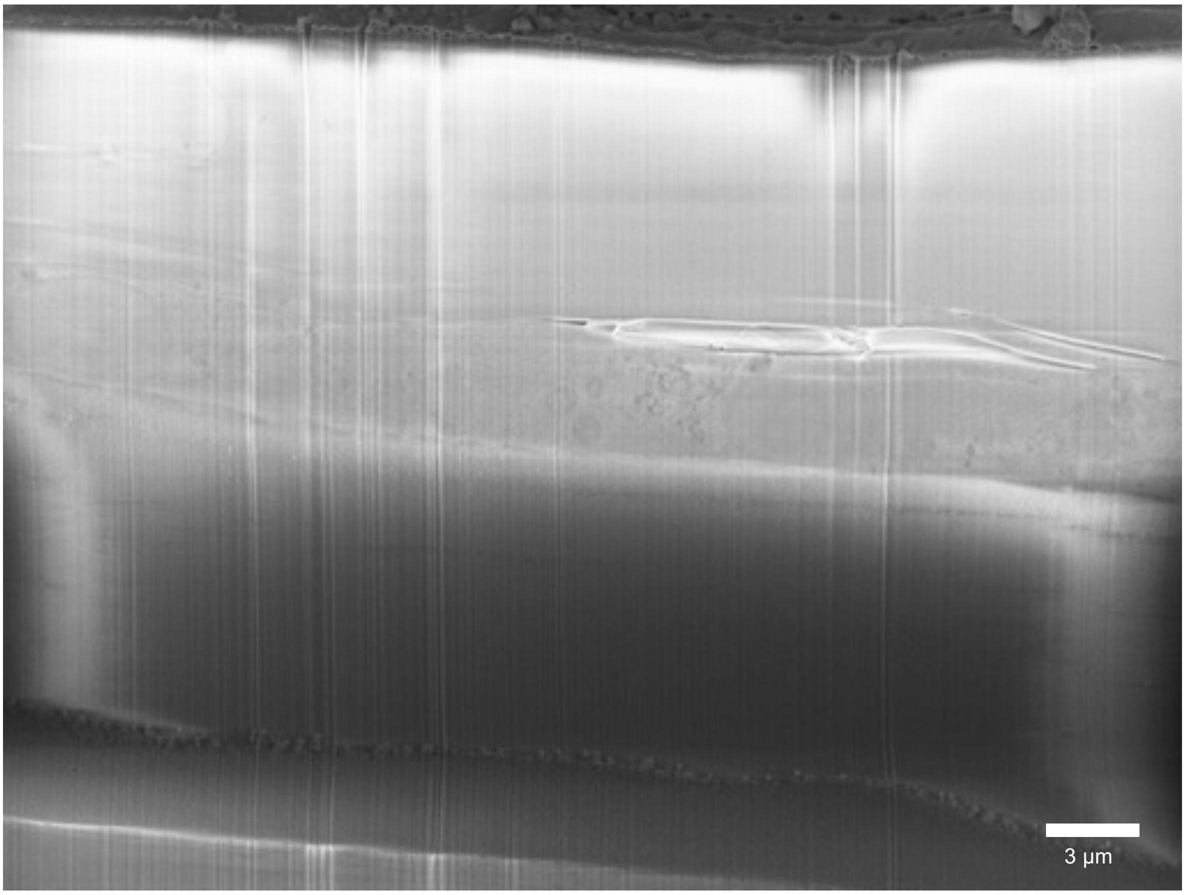

Figure S3. Freeze fracture of scales show the distinct collagen orientations. Recordings of a fractured mature scale using SE2 and BSE. In the left bottom corner, the mineralized tissue is present (pink segmentation). On the right side of the bone, there is collagen present with different orientations.

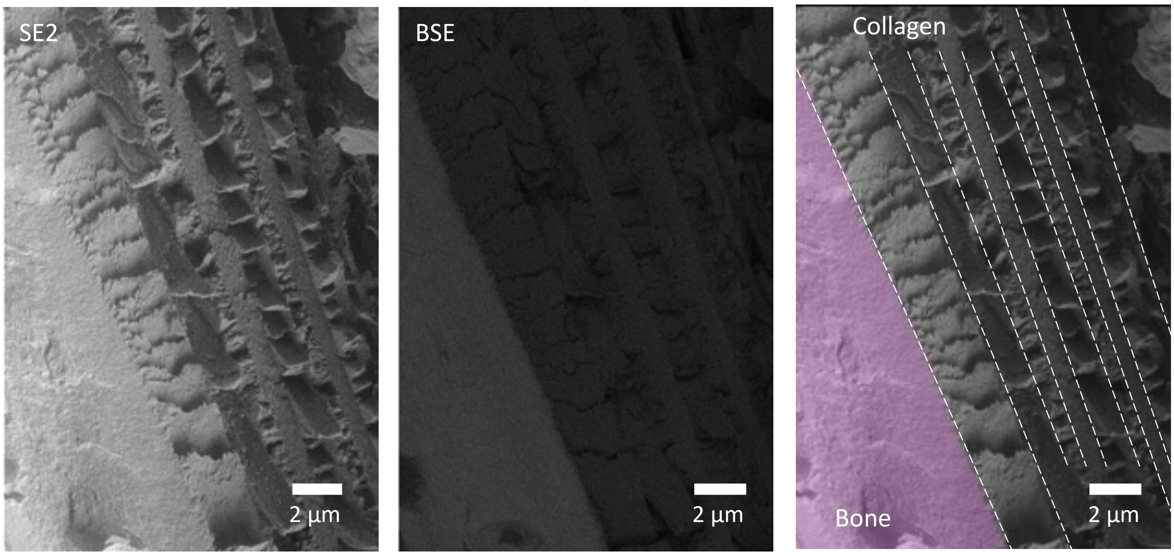

Figure S4. A shift in Z needed to be applied to the nucleus channel (405) to fit the elasmoid cells. Since both stainings (Hoechst and SP7) are expressed in the nucleus, this shift was corrected to get colocalization in the nucleus with both signals. (i) Before the Shift – Z and (ii) after the shift – Z of 4.5 slices (equals 1.6 µm)

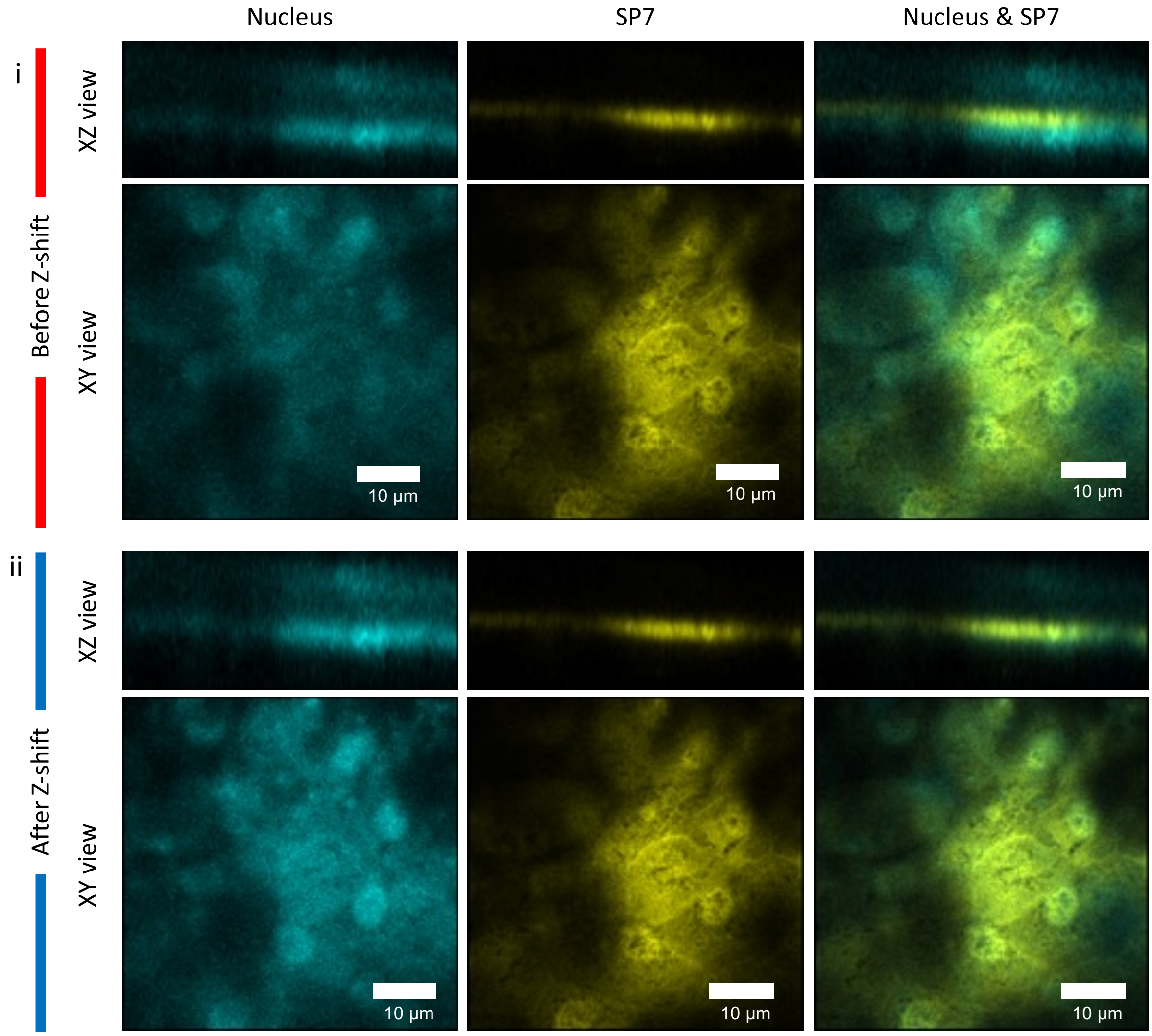

Movie 1. A video of figure 4 shows the zebrafish scale at the cryo-FIB/SEM imaging plane, imaged for 3,5 µm in depth. This movie shows the 3 elasmoblasts lying next to each other, underneath the cryo-protectant dextran solution. The cells are followed in depth by an elasmoidin layer (collagen) and at the bottom there is a mineral layer. During the movie, the main progression can be seen in the cellular organelles, like ER and mitochondria.

The images are recorded with a voxel size of x= 18nm, y=18nm, z=30 nm and afterwards the serial sections were processed and aligned.

Movie 2. A video of figure 4, resliced to match the x-y view in CACM (see Fig. 5), starting at the top until a few micrometers into the elasmoidin layer. This shows the 3 elasmoblasts lying next to each other, underneath the cryo-protectant dextran solution. The cells are followed in depth by an elasmoidin layer (collagen). Here, we can see the different orientations of collagen.
