# Supplementary material for: Precise targeting for 3D cryo-correlative light and electron microscopy volume imaging of tissues using a FinderTOP
Source: Commun Biol. 2023 May 11;6:510. doi: 10.1038/s42003-023-04887-y (PMC10175257; doi:10.1038/s42003-023-04887-y)
Supplement: Supplementary file 3 — Description of Additional Supplementary Data [file 42003_2023_4887_MOESM3_ESM.docx]

**Description of Additional Supplementary Files**

**File name:** Supplementary Movie 1

**Description:** A video of figure 4 shows the zebrafish scale at the cryo-FIB/SEM imaging plane, imaged for 3,5 μm in depth. This movie shows the 3 elasmoblasts lying next to each other, underneath the cryo-protectant dextran solution. The cells are followed in depth by an elasmoidin layer (collagen) and at the bottom there is a mineral layer. During the movie, the main progression can be seen in the cellular organelles, like ER and mitochondria. The images are recorded with a voxel size of x= 18nm, y=18nm, z=30 nm and afterwards the serial sections were processed and aligned..

**File name:** Supplementary Movie 2

**Description:** Description: A video of figure 4, resliced to match the x-y view in CACM (see Fig. 5), starting at the top until a few micrometers into the elasmoidin layer. This shows the 3 elasmoblasts lying next to each other, underneath the cryo-protectant dextran solution. The cells are followed in depth by an elasmoidin layer (collagen). Here, we can see the different orientations of collagen
